# Performance Prediction of a Synchronization Link for Distributed Aerospace Wireless Systems

**DOI:** 10.1155/2013/159742

**Published:** 2013-07-22

**Authors:** Wen-Qin Wang, Huaizong Shao

**Affiliations:** School of Communication & Information Engineering, University of Electronic Science and Technology of China, Chengdu, China

## Abstract

For reasons of stealth and other operational advantages, distributed aerospace wireless systems have received much attention in recent years. In a distributed aerospace wireless system, since the transmitter and receiver placed on separated platforms which use independent master oscillators, there is no cancellation of low-frequency phase noise as in the monostatic cases. Thus, high accurate time and frequency synchronization techniques are required for distributed wireless systems. The use of a dedicated synchronization link to quantify and compensate oscillator frequency instability is investigated in this paper. With the mathematical statistical models of phase noise, closed-form analytic expressions for the synchronization link performance are derived. The possible error contributions including oscillator, phase-locked loop, and receiver noise are quantified. The link synchronization performance is predicted by utilizing the knowledge of the statistical models, system error contributions, and sampling considerations. Simulation results show that effective synchronization error compensation can be achieved by using this dedicated synchronization link.

## 1. Introduction

Distributed aerospace wireless systems have attained more and more interests over the last years as they are seen as a potential means of countering vulnerability to electronics countermeasure [1–7], especially in directional responsive jamming, and avoiding physical attack to the communication platforms [8]. Furthermore, distributed configuration allows a passive receiver teamed with a transmitter at a safe standoff distance. Distributed aerospace wireless systems can be used in many different applications, for example, wireless communications, wireless sensor networks and distributed radars [9–11]. Without loss of generality, this paper considers mainly radar-related applications, especially for the distributed synthetic aperture radar (SAR) imaging. The proposed method is also effective for other distributed aerospace wireless systems.

In distributed radar systems, to measure the echo pulses coherently, the phase information of the transmitted pulse has to be preserved. For a monostatic radar system, in which the colocated transmitter and receiver use the same oscillator, the phase decorrelates over a very short period of time. In contrast, a distributed radar system uses a receiver that is spatially displaced from the transmitter, and hence, the independent phase noise of the transmitter and receiver oscillators does not cancel out. This superimposed phase noise corrupts the received signal over the whole coherent integration time, and therewith severely compromises the subsequent radar performance.

Although the feasibility of distributed radar system concept was already demonstrated by experimental investigations in [12–15], the time and frequency synchronization aspects are still impediments to distributed radar system development [16–20]. The requirement of phase stability for distributed radar system was discussed in [16]. The impact of limited oscillator stability in bi- and multistatic SAR was discussed in [21], which pointed out that uncompensated phase noise may cause a time variant shift, spurious sidelobes, and a deterioration of the impulse response, as well as a low-frequency phase modulation on the received signal. An estimation of oscillator's phase offset in bistatic interferometry SAR was investigated in [22]. In practice, time synchronization is also required for data acquisition. The linear and random time synchronization errors were discussed in [23]; a conclusion was made that linear frequency synchronization errors would lead to a lower imaging resolution and a movement of the target image.

In [24], we investigated a direct-path signal-based technique to compensate the oscillator phase noise for distributed radar systems. The direct-path signal of the transmitter is received with an appropriative antenna divided into two channels. One is passed through an envelope detector and used to synchronize the sampling clock, and the other one is down-converted and used to compensate the phase synchronization errors. However, this approach can be applied only in a limited observation region. The use of continuous duplex intersatellite links for oscillator drift is proposed in [25] and further investigated in [26]. However, this approach destroys the passive characteristic of the receiver and increases its vulnerability, which greatly limits the application scope of the distributed radar system. To get around this disadvantage, we extend the approach to general distributed radar system. The use of a dedicated synchronization link to quantify and compensate the carrier frequency instability is proposed. With the analytical models of phase noise, the possible synchronization accuracy, which may be impacted by oscillator, phase-locked loop, and receiver noise, is quantified. This work can provide a reference to develop practical time and frequency synchronization for distributed radar systems.

The remaining sections of this paper are organized as follows. The time and frequency synchronization scheme via a dedicated microwave communication link is proposed in Section 2. With the analytical models described in Section 3, the time and frequency synchronization accuracy is predicated in Section 4. Finally, Section 5 concludes the whole paper.

## 2. Synchronization Schemes

**Figure 1:**
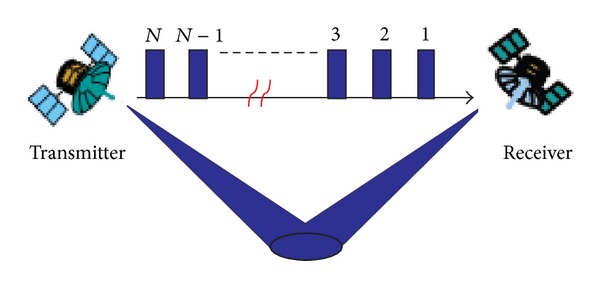
Model of the time and frequency synchronization link.

Depending on the hardware and affordable synchronization system complexity, various hardware configurations can be employed to establish the synchronization link. As mentioned previously, the duplex intersatellite link [25] demolishes the passive characteristic of receiver and increases its vulnerability, and the direct-path signal-based approach [24] limits the observation region. To overcome these disadvantages, in this paper, we investigate a monodirection synchronization link, as shown in Figure 1.

According to the synchronization schemes, the transmitter repeatedly transmits a synchronization signal, which is a linearly frequency modulated (LFM) signal. The frequency of the oscillator in the transmitter at the start of data take  *t*_0_  is  *f*_*i*_ = *f*_0_ + Δ*f*_*i*_, with a nominal frequency  *f*_0_  and a frequency offset  Δ*f*_*i*_. The phase *ϕ*_*T*_(*t*)  at time *t* is the integration over frequency [26]:

(1) $$\begin{array}{c} \phi_{T}\left(t\right)=2\pi \int_{t_{0}}^{t}f_{T}\left(t\right)dt+\varphi_{Ti}+n_{\varphi T}\left(t\right), \end{array}$$

where  *φ*_*Ti*_  is the initial phase and  *n*_*φT*_(*t*)  is the oscillator phase noise.

The receiver receives the signal after a delay *τ*_*i*_ corresponding to the time it takes the signal to travel the transmitter-to-receiver distance  *r*. At the receive instance  *t* + *τ*_*i*_, the phase *ϕ*_*R*_(*t* + *τ*_*i*_)  of the oscillator in receiver is

(2) $$\begin{array}{c} \phi_{R}\left(t+\tau_{i}\right)=2\pi \int_{t_{0}}^{t+\tau_{i}}f_{R}\left(t\right)dt+\varphi_{Ri}+n_{\varphi R}\left(t+\tau_{i}\right). \end{array}$$

The demodulated phase available at receiver is the difference between (1) and (2) after including the system and path contributions. This phase difference can be used to obtain the compensation phase. Note that, here, *t*_0_ can be set to zero without restricting generality.

A practical problem is to decide the synchronization repeatedly frequency *f*_syn_, carrier frequency *f*_0_, and pulse duration *T*_*p*_. Additionally, the changes of propagation conditions will result in amplitude and phase fluctuations. Furthermore, an estimate of the time-continuous compensation phase must be recovered from the discrete samples (e.g., *sinc* interpolation). Therefore, the synchronization accuracy must be predicted, and its feasibility must be evaluated prior to developing this synchronization system. In the following, we focus on deriving quantitative estimations for predicting the performance of this synchronization link.

## 3. Modeling Frequency Instability in Distributed Radar Systems

### 3.1. Model of Reference Oscillator Frequency Instability

Generally, the performance of oscillator instability is evaluated with Allan variance [27] in time domain or phase noise power spectral density (PSD)  *S*_*φ*_(*f*)  in frequency domain [28]. Although an oscillator's phase noise is a complex interaction of variables, ranging from its atomic composition to the physical environment of the oscillator, in the condition that the phase fluctuations occurring at rates  *f*  and are small compared with one radian, a good approximation is [29]

(3) $$\begin{array}{c} S_{\varphi }\left(f\right)=a\cdot f^{-4}+b\cdot f^{-3}+c\cdot f^{-2}+d\cdot f^{-1}+e, \end{array}$$

where the coefficients  *a*  to  *e*  describe the contributions from (a) random walk frequency noise, (b) frequency flicker noise, (c) white frequency noise, (d) flicker phase noise, and (e) white phase noise, respectively.

One cannot foresee to predict the synchronization accuracy without a model of phase instability. Unfortunately, the frequency-domain expression  *S*_*φ*_(*f*)  cannot be directly used in distributed radar systems. The white noise model cannot describe the statistical process of phase noise. The Wiener noise model [30] cannot describe the low-frequency phase noises which are of great interest for distributed radar system. Hence, we use a time-domain analytical model of reference oscillator phase noise. This model may represent the output signal of a hypothetical filter with impulse response  *h*(*t*)  receiving an input signal  *x*(*t*).

The spectral density of the output signal is given by the product  *S*_*x*_(*f*) | *H*_*φ*_(*f*)|^2^, where the filter transfer function  *H*_*φ*_(*f*)  is the Fourier transform of  *h*_*φ*_(*t*). Note that, here, the | *H*_*φ*_(*f*)|^2^  must be satisfied with

(4) $$\begin{array}{c} {\left|H_{\varphi }\left(f\right)\right|}^{2}=\{\begin{array}{cc} S_{\varphi }\left(f\right), & f_{l}\leq \left|f\right|\leq f_{h},\\ S_{\varphi }\left(f_{l}\right), & \left|f\right|\leq f_{l},\\ 0, & \text{otherwise}, \end{array} \end{array}$$

where a sharp-up cutoff frequency  *f*_*h*_  and a sharp-down cutoff frequency  *f*_*l*_  are introduced. Note that time domain stability measures sometimes depend on  *f*_*h*_  and  *f*_*l*_  which should be given to obtain numerical results. In this paper,  *f*_*h*_ = 5 kHz and  *f*_*l*_ = 0.01 Hz are assumed.

The phase noise in time domain can then be represented by

(5) $$\begin{array}{c} \varphi_{\text{osc}}\left(t\right)=\sqrt{K}x\left(t\right)⊗h\left(t\right), \end{array}$$

where *K* is a constant,  *φ*(*t*)  denotes the phase noise sequence in time domain and ⊗ denotes the convolution operator.

### 3.2. Model of Phase-Locked Loop (PLL) Phase Noise

**Figure 2:**
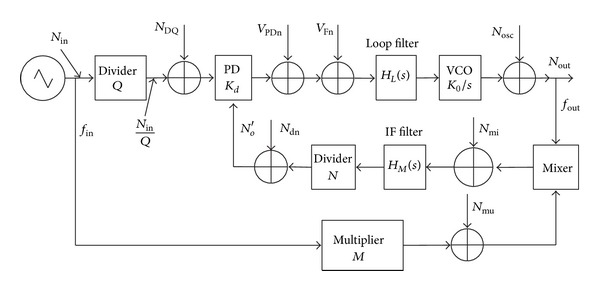
Model of a general PLL with additive noise.

Figure 2 shows a fairly general PLL arrangement with a phase detector (PD), a low-pass loop filter  *H*_*L*_(*s*), a voltage controlled oscillator (VCO) in the forward path and a mixer, an intermediate frequency (IF) filter  *H*_*M*_(*s*), and a divider (÷*N*). Additionally, a divider (÷*Q*) and a multiplier (×*N*) are also placed. Since all the noises generated or added in individual PLL blocks are small compared with the useful signals, the small signal theory makes it possible to use the Laplace transform to find the output noise of the considered PLL system or, more exactly, the, respectively, power spectral densities.

According to Figure 2, we can get that [31]

(6) NPLL(s) =[Nin(s)(M+NQ1FM(s))   +(NDQ(s)−Ndn(s)+VPDn(s)+VFn(s)Kd)NFM(s)  +Nmu(s)−Nmi(s)]·H(s)+Nosc[1−H(s)],

where the effective loop transfer function  *H*(*s*)  is

(7) $$\begin{array}{c} H\left(s\right)=\frac{K_{0}K_{d}F\left(s\right)}{s+K_{0}K_{d}F\left(s\right)}, \end{array}$$

with

(8) $$\begin{array}{c} F\left(s\right)=\frac{1+s\tau_{2}}{s\tau_{1}}. \end{array}$$

The  *τ*_1_  and  *τ*_2_  are the loop low-pass filter parameters. The details can be found in [32]. Note that all the other variables are illustrated in Figure 2. Since most of the noise components are random and uncorrelated, the power spectral density of the PLL output phase noise is

(9) Sφ,PLL(f) ={Sφ,in(f)(M+NQ)2   +[Sφ,DQ(f)+Sφ,dn(f)+Sφ,PDn(f)+Sφ,Fn(f)Kd2]   ×N2+Sφ,mu(f)+Sφ,mi(f)}  ·|H(jf)|2+Sφ,osc(f)|1−H(jf)|2.

We see that the first term in the brace of (9) is inevitable since it is merely a multiplied reference oscillator noise. The second term includes the divider noise, phase detector noise, and loop filter noise, all multiplied by the division ratio *N*. Finally, with the third term, the multiplier and mixer noises are added; generally, they are small compared with the second term [33]. Hence, all the additive noises, due to the phase detector, loop frequency divider, loop amplifiers, and loop filters are required to quantify prior to predicting the synchronization accuracy.

#### 3.2.1. Phase Detector and Mixer

 There are both theoretical and experimental lines of evidences that additive noise due to the mixers is quite small and of the order of the loading circuit noise. Experimental results show that the best phase detector is a double-balanced mixer [28]. Measurements reveal that the phase noise in a double-balanced mixer can be approximated as [34]

(10) $$\begin{array}{c} S_{\varphi ,\text{PDn}}\left(f\right)\approx \frac{10^{-14\pm 1}}{f}+10^{-17} \end{array}$$

and for phase detector based on CMOS logic family is

(11) $$\begin{array}{c} S_{\varphi ,\text{PDn}}\left(f\right)\approx \frac{10^{-12.7}}{f}+10^{-16.2}. \end{array}$$

#### 3.2.2. Frequency Divider

 Theoretically, the division process reduces the input PSD in proportion to the square of the division factor  *N* ^2^. However, investigation of the divider output phase noise performed by Kroupa [35] reveals that the output phase noise is

(12) Sφ,dn(f)≈Sφ,dn,in(f)N2+10−10±1+10−27±1f02f+10−16±1+10−22±1f0.

#### 3.2.3. Frequency Multiplier

 The phase noise PSD at the output of a frequency multiplier is equal to its input multiplied by the square of multiplication factor plus an additive term, that is,

(13) Sφ,mu(f)≈Sφ,mu,in(f)·N2+10−13±2f+10−16±1.

#### 3.2.4. Amplifier

 The output phase noise in the low-frequency operational amplifier implemented with GaAs/GaA1As heterojunction bipolar transistors is [36]

(14) $$\begin{array}{c} S_{\varphi ,\text{amp},\text{IF}}\left(f\right)\approx \frac{10^{-13}}{f}. \end{array}$$

For radio frequency (RF) amplifier noise, generally, only a narrow bandwidth around the carrier is considered; one-half of the thermal white noise contributes to the amplitude noise modulation and the other half to the phase noise modulation. Hence, a theory limit of the phase noise at the RF amplifier output is [36]

(15) $$\begin{array}{c} S_{\varphi ,\text{amp},\text{RF}}\left(f\right)\approx 4\frac{kTR}{V_{\text{rms}}^{2}}, \end{array}$$

where  *k*  and  *T*  are Boltzmann's constant and temperature in Kelvin, respectively.

#### 3.2.5. VCO

 The VCO phase noise improves as it goes to farther offsets from the carrier. Although there could be more regions with different slopes to the phase noise, a reasonable model for this is to divide this noise into three regions. A fairly general VCO phase noise equation is [37]

(16) Sφ,vco(f)≈f02·10−11.6f3·QL2+f02·10−15.6f2·QL2+10−11f·QL2+10−15,

where  *Q*_*L*_  is the loaded quality factor of the oscillator.

#### 3.2.6. Loop Filter

 As one of the most important parts in the PLL synthesizer, loop filter has various topologies. For distributed radar systems, passive filters are generally recommended, because they have the advantages of lower cost and no active devices to add noise. Moreover, to reduce spur levels, a fourth-order filter is used in this paper, because fourth order and higher-order filters become more practical when the spurs to be filtered are at least 20 times the loop bandwidth [32]. As all resistors create thermal noise, there are two major sources of noise, namely, some types of capacitors and resistors. Typically, the contribution from this resistor noise within the loop bandwidth is negligible. In the case of a resistor, this noise voltage is the thermal noise generated by the resistor. We then have

(17) $$\begin{array}{c} R_{\text{noise}}\left(R\right)=\sqrt{4TkR}, \end{array}$$

where the units are$\;\text{V}/\sqrt{\text{Hz}}$. Since phase noise is normalized to a 1 Hz bandwidth, one can disregard the denominator and consider the units to be in Volts.

### 3.3. Model of Receiver Noise

 The receiver noise, consisting of thermal noise and the noise collected by the antenna, will introduce both amplitude and phase fluctuation to the synchronization signal. Here, only phase fluctuation is considered; its influence on the signal phase is described by the receiver phase noise spectral density function  *S*_*φ*,SNR_(*f*). For band-limited white Gaussian noise, the PSD of phase noise is related to the SNR (signal-to-noise ratio) through [38]

(18) $$\begin{array}{c} S_{\varphi ,\text{SNR}}\left(f\right)\approx \frac{1}{2B_{w}\cdot \text{SNR}}, \end{array}$$

with the receiver (noise) bandwidth  *B*_*w*_. Note that, the following matched filtering will further improve the receiver SNR.

### 3.4. Model of Distributed Radar System Frequency Instability

 Since only phase noise is of great interest, the modulation waveform used for range resolution can be ignored, and the distributed radar system can be simplified to an “azimuth only” system [16]. Suppose that the transmitted signal is sinusoid whose phase argument is

(19) $$\begin{array}{c} s_{T}\left(t\right)=2\pi f_{T}t+\varphi_{T}\left(t\right). \end{array}$$

The first term is the carrier frequency, and the second represents the phase deviations from the error-free carrier which includes the sum of phase noises discussed previously, that is,

(20) $$\begin{array}{c} \varphi_{T}\left(t\right)=\varphi_{\text{sum}}\left(t\right)=\varphi_{\text{osc}}\left(t\right)+\varphi_{\text{PLL}}\left(t\right)+\varphi_{\text{SNR}}\left(t\right). \end{array}$$

After reflection from a target, the received signal phase is that of the transmitted signal delayed by the round-trip time  *τ*. The receiver output signal phase$\;\hat{s}(t)$ results from demodulating the received signal with the receiver oscillator which has the same form as the transmitter oscillator:

(21) $$\begin{array}{c} s_{R}\left(t\right)=2\pi f_{R}t+\varphi_{R}\left(t\right). \end{array}$$

Hence, we have

(22) $$\begin{array}{c} \hat{s}\left(t\right)=2\pi \left(f_{R}-f_{T}\right)t+2\pi f_{T}\tau +\varphi_{R}\left(t\right)-\varphi_{T}\left(t\right). \end{array}$$

The first term is a frequency offset arising from the nonidentical oscillator frequencies. It is not important and can be ignored. The second term forms the usual Doppler term as round-trip time to the target varies it should be preserved. The last two terms represent the frequency synchronization errors which are of interest for extracting the frequency synchronization errors; hence, the phase errors in distributed radar system can be modeled as

(23) $$\begin{array}{c} \phi_{B}\left(t\right)=\varphi_{T}\left(t\right)-\varphi_{R}\left(t\right). \end{array}$$

It is assumed that  *φ*_*T*_(*t*)  and  *φ*_*R*_(*t*)  are independent random variables having identical PSD  *S*_*φ*,sum_(*f*); then, the phase synchronization error PSD in distributed radar system is

(24) $$\begin{array}{c} S_{\varphi_{B}}\left(f\right)=2S_{\varphi ,\text{sum}}\left(f\right), \end{array}$$

where the factor *2* arises from the use of two independent oscillators.

## 4. Prediction of Link Synchronization Accuracy

The synchronization signals must be sufficiently decoupled from the radar signals; otherwise, they may cause problems when using the same carrier frequency. Hence, we suppose the phase arguments of synchronization signal and normal radar signal are given, respectively, by

(25) $$\begin{array}{c} s_{T,\text{syn}}\left(t\right)=2\pi f_{T,\text{syn}}t+\varphi_{T,\text{syn}}\left(t\right),\\ s_{T,\text{sar}}\left(t\right)=2\pi f_{T,\text{sar}}t+\varphi_{T,\text{sar}}\left(t\right). \end{array}$$

Similarly, the first term is the error-free carrier frequency, and the second represents the phase deviations from the error-free carrier. In the same manner like (23), we get that

(26) $$\begin{array}{c} \phi_{\text{syn}}\left(t\right)=\varphi_{T,\text{syn}}\left(t\right)-\varphi_{R,\text{syn}}\left(t\right),\\ \phi_{\text{sar}}\left(t\right)=\varphi_{T,\text{sar}}\left(t\right)-\varphi_{R,\text{sar}}\left(t\right). \end{array}$$

With the proposed synchronization compensation method, the phase compensation term is *ϕ*_syn_(*t*) · *f*_*T*,sar_/*f*_*T*,syn_. Thus, the synchronization accuracy is decided by

(27) $$\begin{array}{c} \phi_{\mathrm{err}}\left(t\right)=\phi_{\text{sar}}\left(t\right)-\phi_{\text{syn}}\left(t\right)\cdot \frac{f_{T,\text{sar}}}{f_{T,\text{syn}}}. \end{array}$$

**Figure 3:**
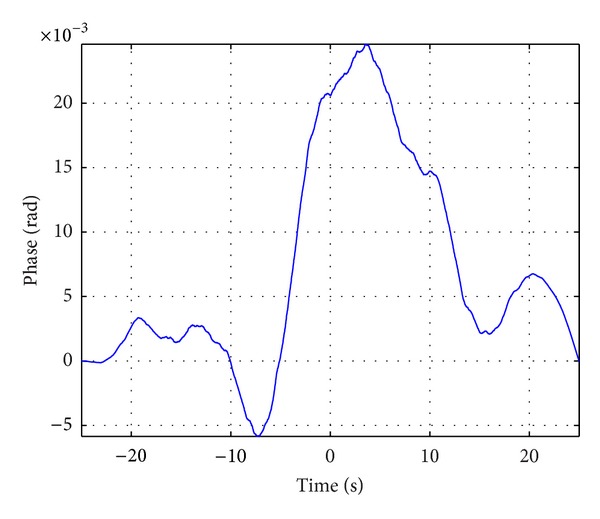
Prediction of phase noise in a typical 10 MHz crystal oscillator.

**Figure 4:**
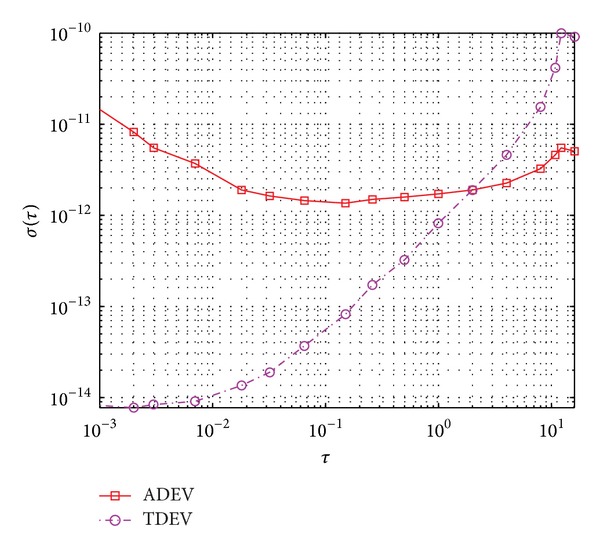
Prediction of frequency instability, ADEV: overlapping Allan standard deviations, TDEV: Allan time standard deviations.

To derive quantitative estimation, we take a typical crystal oscillator, shown in Table 1, as an example. With the model of reference oscillator frequency instability, simulated phase errors in this 10 MHz oscillator are shown in Figure 3 for a time interval of 50 seconds. From its Allan variance shown in Figure 4, we can see that this oscillator can be regarded as a representative example for the ultrastable oscillator of current aerospace radar systems. Note that Figure 3 is the result of one typical simulation, and Figure 4 is the statistical result of many simulations.

**Figure 5:**
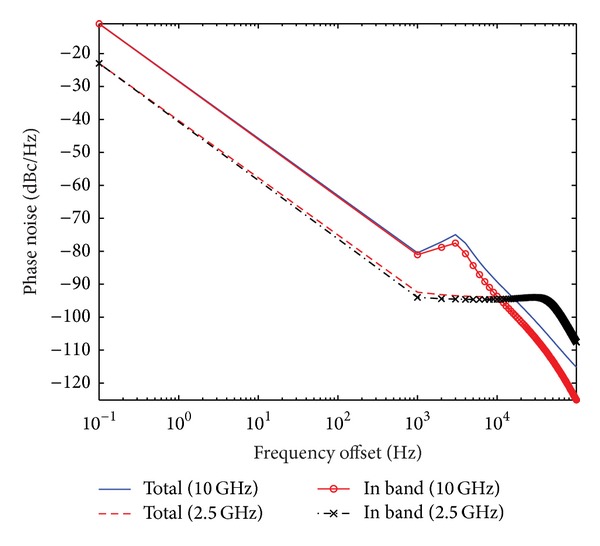
Comparative results of output phase noise between synchronization channel and radar channel.

From the models of phase noise described in the previous sections, we get the comparative results of the output phase noise between the radar channel and synchronization channel, shown in Figure 5. The observation could be made that, within the loop bandwidth, the PLL phase detector is typically the dominant noise source, and outside the loop bandwidth, the VCO noise is often the dominant noise source. Hence, the performance of the synchronization link will be impacted by the common misconception that the phase noise will vary with 20log⁡(*f*_0_/*f*_osc_) with *f*_osc_ being the oscillator frequency.

Additionally, the phase of the synchronization link may be influenced by receiver noise, analog-to-digital convertor (ADC), and data interpolation. As we have mentioned previously, the receiver noise determined by SNR is of special interest. Furthermore, the synchronization phase is sampled, which requires a later interpolation of the compensation phase. We may choose to filter the compensation phase with an arbitrary transfer function *H*_syn_(*f*) like [26]. Note that, if distributed SAR imaging is considered, the compensated SAR phase (SAR phase after subtracting the compensation phase) is filtered through azimuth compression. This filter is described by the transfer function *H*_az_(*f*) and is dependent on the azimuth processing. The impact of receiver noise on synchronization phase is [26]

(28) σSNR2=12fsyn·SNR×∫−fsyn/2fsyn/2Sφ,SNR(f)|Hsyn(f)Haz(f)|2df,

where  *f*_syn_  represents the synchronization repeatedly frequency rate.

In the case of digital-to-analog convertor (DAC), the quantization errors result in what appears to be a white noise floor but is actually a “sea” of very finely spaced discrete spurs. For a *N*-bit DAC, the phase errors due to quantization errors are determined by [39]

(29) $$\begin{array}{c} \varphi_{\max}\approx \arctan\left(\frac{1}{2^{N}-1}\right). \end{array}$$

Note that *N* = 12 is assumed in the following simulation.

The interpolation error is because frequency components outside the range −*f*_syn_/2 < *f* < *f*_syn_/2 are lost due to the sampling and hence cannot be reconstructed. The interpolation variance is [21]

(30) $$\begin{array}{c} \varphi_{\mathrm{int}}^{2}=2{\left(\frac{f_{0}}{f_{\text{osc}}}\right)}^{2}\int_{f_{\text{syn}}/2}^{\infty }{\left|H_{\text{az}}\left(f\right)\right|}^{2}df. \end{array}$$

The factor *2* is due to the use of two independent oscillators, and the scaling factor in the parentheses is due to the multiplication of the oscillator frequency  *f*_osc_  with (*f*_0_/*f*_osc_) to obtain the radar signal with carrier frequency  *f*_0_.

**Figure 6:**
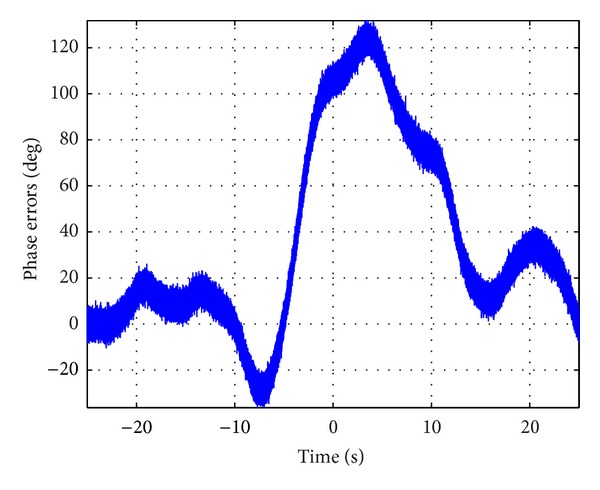
Prediction of phase errors via the dedicated synchronization link.

**Figure 7:**
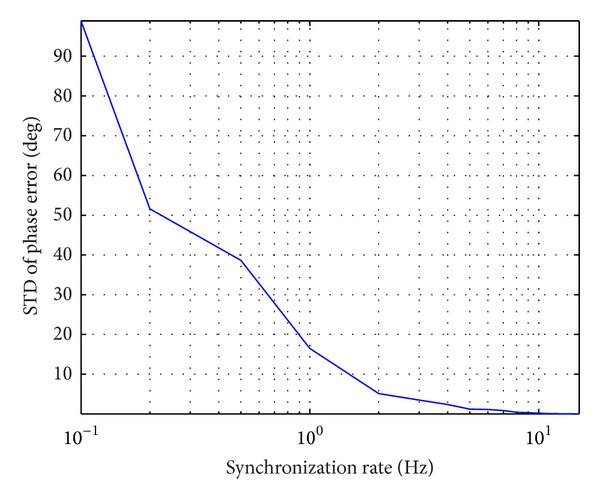
Prediction of phase synchronization accuracy versus synchronization repeatedly frequency rate.

Finally, further suppose that the signal bandwidths of radar signal and synchronization signal are 100 MHz and 1 GHz, respectively.  Figure 6 shows the prediction of total phase errors contributed by the synchronization link. The prediction of standard deviation (STD) of the phase synchronization error contributed by synchronization link versus synchronization rate is shown in Figure 7. Note that Figure 7 is a statistical result with twenty realizations of the stochastic process described previously. From Figure 7, we can see that successful synchronization error compensation is possible by using this dedicated synchronization link with enough synchronization rate.

## 5. Conclusion

A dedicated synchronization link is a solution to avert the performance degradation due to oscillator frequency instability in distributed radar system. Hence, the use of a dedicated synchronization link to quantify and compensate oscillator frequency instability is investigated in this paper. With analytical models of phase noise, closed analytic expressions for the link performance are derived. We utilize the knowledge of statistical models, system error contributions, sampling considerations, and signal processing parameters to investigate the residual phase error after synchronization, and the possible error contributions including oscillator, PLL, and receiver noise are quantified. Simulation results show that effective synchronization error compensation is possible by using this dedicated synchronization link. Note that this paper considers mainly radar-related synchronization applications, but the presented method and analysis results are also effective for other distributed wireless systems.

## Figures and Tables

**Table 1:** Phase noise parameters of one typical crystal oscillator.

Frequency offset (Hz)	1	10	100	1 k	10 k
Phase noise PSD (dBc/Hz)	−80	−100	−130	−150	−160
